# Effect of the bivalent HPV vaccine on viral load of vaccine and non-vaccine HPV types in incident clearing and persistent infections in young Dutch females

**DOI:** 10.1371/journal.pone.0212927

**Published:** 2019-03-04

**Authors:** Pascal van der Weele, Martijn Breeuwsma, Robine Donken, Elske van Logchem, Naomi van Marm-Wattimena, Hester de Melker, Chris J. L. M. Meijer, Audrey J. King

**Affiliations:** 1 National Institute for Public Health and the Environment (RIVM), Centre for Infectious Disease Control, Bilthoven, the Netherlands; 2 Vrije Universiteit - University Medical Center (VUmc), Department of Pathology, Amsterdam, the Netherlands; Rudjer Boskovic Institute, CROATIA

## Abstract

**Background:**

HPV vaccination with the bivalent vaccine is efficacious against HPV16 and 18 infections and cross-protection against non-vaccine HPV types has been demonstrated. Here, we assessed (cross-) protective effects of the bivalent HPV16/18 vaccine on incident and persistent infections and viral load (VL) of fifteen HPV types in an observational cohort study monitoring HPV vaccine effects.

**Methods:**

Vaginal samples were obtained annually. Type-specific VL assays were developed for HPV6,11,31 33,35,39,45,51,52,56,58,59 and 66 and used in addition to existing HPV16 and 18 assays. Rate differences of incident clearing and persistent infections were correlated with differences in VL and vaccination status.

**Results:**

HPV16/18 vaccination resulted in significantly lower incidence of HPV16/18 infections and significantly lower VL in breakthrough HPV16 (p<0.01) and 18 infections (p<0.01). The effects of vaccination on non-vaccine type VL were ambiguous. Incidence and/or persistence rates of HPV31, 33, 35 and 45 were reduced in the vaccinated group. However, no significant type specific VL effects were found against HPV31, 33, 45, 52 in the vaccinated group. For HPV 6, 59 and 66 no significant reductions in numbers of incident and persistent infections were found, however borderline) VL reductions following vaccination were observed for HPV6 (p = 0.01), 59 (p = 0.10) and 66 (p = 0.03), suggesting a minor effect of the vaccine on the VL level of these HPV types. Overall, vaccination resulted in infections with slightly lower VL, irrespective of HPV type.

**Conclusions:**

In conclusion, vaccination with the bivalent HPV16/18 vaccine results in significantly reduced numbers of HPV16 and 18 incidence rates and reduced VL in breakthrough infections. Significant reductions in incident and/or persistent HPV31, 33, 35 and 45 infections were found, but no significant effect was observed on the VL for infections with these types. For the other non-vaccine HPV types no reduction in incident and/or persistent infections were found, but overall the VL tended to be somewhat lower in vaccinated women.

## Introduction

Cervical intraepithelial lesions (CIN) and cervical cancer are caused by persistent high-risk human papillomavirus (hrHPV) infections [1]. Progression from initial infection to precancerous lesions and ultimately cervical cancer can take decades [2, 3]. CIN2 and CIN3 are the most advanced precursor lesions for cervical cancer and are accepted by the WHO as surrogate cervical cancer endpoint markers for vaccine efficacy. Later, persistent HPV infections (>6 months) were also accepted as adequate surrogate endpoint markers [4]. Among hrHPV types, HPV16 and 18 cause approximately 70% of all cervical cancers worldwide. A further 20% of the worldwide cervical cancer burden is added by HPV31, 33, 45, 52 and 58 [5].

To prevent HPV-associated malignancies, three highly efficacious prophylactic recombinant vaccines are currently registered and commercially available. The bivalent vaccine protects against HPV16 and 18, quadrivalent adds HPV6 and 11, and nonavalent further adds HPV31, 33, 45, 52 and 58 [6]. Varying degrees of cross-protection have been described against non-vaccine oncogenic HPV types for the bivalent and the quadrivalent vaccines [7–15]. The bivalent vaccine does seem to generate a broader cross-protective response against non-vaccine HPV types associated with CIN2+ (HPV31, 33, 39, 45 and 51) than the quadrivalent vaccine (limited to HPV31) [16]. Interestingly, limited VE has also been reported for the bivalent vaccine against HPV6 and 11 infections [17], resulting in a reduced occurrence of anogenital warts in England [18, 19]. However, these results should be further confirmed. Cross-protection is currently a heavy focus of investigation in the field and cross-protective effects are being (partly) attributed to the AS04 adjuvant (3-*O*-desacyl-4′-monophosphoryl lipid A adsorbed on aluminum salt), which appears to induce enhanced and longer-lasting antibody titers than the aluminum hydroxyphosphate sulfate adjuvant used in the quadri- and nonavalent vaccines [20]. Vaccination with the bivalent HPV vaccine also offers high, but not complete, protection against vaccine type incident infections [21, 22].

The protective effect against persistent HPV16/18 and some non-vaccine type HPV infections and associated CIN2+ lesions [11, 12, 16, 21–24], combined with the lower VE against incident HPV16/18 infections [21, 22], suggest that infections that establish in vaccinated individuals are very unlikely to persist. It has been previously described that persistence of HPV infection is associated with high viral load (VL) [25, 26]. Lower VL of these HPV types could be expected among HPV infections in vaccinated compared to non-vaccinated individuals. Consequently, longitudinal VL measurements might give additional insights in vaccine effects.

In 2009, bivalent HPV vaccination was introduced in the Netherlands. At the same time, the HPV Amongst Vaccinated and Non-vaccinated Adolescents (HAVANA) cohort study was started to monitor vaccine effectiveness [12, 27, 28]. Recently, detailed analyses of this cohort were published, including type-specific and pooled vaccine effectiveness estimates against incident and persistent infections. A high vaccine effectiveness against HPV16 and HPV18 infections was shown, as well as significant cross-protection against HPV31, 33 and 45 [12]. The present analysis aimed to use the same cohort study to assess type-specific (TS) incident and persistent HPV infections and to compare their VLs in both vaccinated and non-vaccinated women in order to see whether TS VL measurements could be used as a marker for HPV type protection.

## Materials and methods

### Study population and HPV DNA genotyping

Vaginal self-samples were obtained annually from participants of the “*HPV Amongst Vaccinated and Non-vaccinated Adolescents*” (HAVANA) observational cohort study, which has been described previously [28]. In short, 29,162 girls, aged 14–16 years old, eligible for catch-up vaccination, were invited for participation in 2009 and 2010. From the invitees, 6% consented to study participation. The inclusion criteria for the present analysis were girls having received either three doses of the bivalent HPV vaccine, or zero doses. Study characteristics for the present population were compared to a previous analysis of this study cohort [12]. Informed consent was obtained from study participants and both parents (if possible), or a legal representative. This study was approved by the Medical Ethics Committee of VUmc Amsterdam (2009/22). For the present analysis, a baseline and seven follow-up samples were available per study participant.

Total DNA was isolated from 200μl of self-samples using the MagNAPure96 platform (Roche Diagnostics) and eluted in 100μl elution buffer. A phocine herpesvirus-1 spike was added to each sample as an internal control for DNA isolation. Isolated DNA was genotyped using the analytically sensitive SPF10-DEIA-LiPA_25_ platform [29–31].

### Plasmid transformation, isolation and quantification

Ampicillin resistant plasmids for HPV6, 11, 31, 33, 35, 39, 45, 51, 52, 56, 58, 59 and 66 were kindly provided by the HPV Reference Center, Karolinska Institutet, Sweden. Plasmids were transformed into heat-competent NEB 5-alpha *E*. *coli* (High Efficiency) (New England Biolabs) according to the manufacturer’s protocol and the cell suspension was subsequently plated on MH agar plates containing ampicillin. Plates were incubated overnight at 37°C and single colonies were transferred to liquid LB medium containing ampicillin. Cultures were grown while shaking to OD_600_ = 2–3, and then centrifuged at 5000g for ten minutes. Plasmid was isolated from cell pellets using GeneJet Plasmid Midiprep kit (Thermo Scientific) according to the manufacturer’s protocol. Isolated plasmid was eluted in 40μl elution buffer and quantitated in triplicate in three individual experiments to minimize assay variation using PicoGreen dsDNA quantification assay (ThermoFisher).

### Assay performance and viral load quantification

New quantitative VL assays for HPV6, 11, 31, 33, 35, 39, 45, 51, 52, 56, 58, 59 and 66 were developed in line with HPV16/18 assays described previously [26]. Primer and probe sequences are shown in S1 Table. Quantifications were performed on the LightCycler480 (Roche Diagnostics) platform. Calibration curves were generated for individual assays using triple measurements of twofold dilution series as described previously [26]. Detection limits were set at the lowest concentration detectable in the linear spectrum of the calibration curve (S1 Table). Assay specificity was determined by testing TS reagents with different plasmids at high concentrations (> 1x10^7^ genome equivalents (GEq)/reaction). To assess possible over- and underscoring of assays, nine dilutions from each calibration curve were tested in the presence and absence of high concentrations (>1.7x10^5^ GEq/reaction) of the eight most prevalent hrHPV types in this study; HPV16, 18, 31, 33, 45, 51, 52 and 58. All measurements were performed in singleplex for optimal sensitivity and normalized by measuring cellular content via beta-actin qPCR [32]. Samples TS HPV DNA positive by genotyping, but without measurable VL were assigned an arbitrary VL of 10^−5^ copies/cell [33].

### Infection and incidence rate definitions

To be included in the analysis of any HPV type, study participants had to be genotyping negative at baseline for that specific HPV type. HPV (baseline) positivity was assessed on a per type basis. Study participants could be included independently in multiple TS analyses. An exclusion for any one HPV type did not result in *de facto* exclusion for other HPV types.

Infection definitions are explained in Table 1. Any TS HPV measurement following a TS HPV negative baseline was considered an incident infection. Clearing infections were defined as a single HPV TS positive measurement with no new HPV TS positive measurement identified within two years (Table 1C–1E). Infection persistence was defined as at least two subsequent HPV TS positive measurements (±12 months between samples; Table 1G–1J). If a study participant fulfilled both the criteria for a clearing and a persistent infection for a single HPV type, only the first infection was included (Table 1F and 1K). For VL analysis of persistent infections, the first positive samples were quantified, since load at first detection is a major determinant of HPV persistence [34].

**Table 1:** Infection definitions as used for this study.

	Baseline TS HPV	Per round (annual) TS HPV					Action taken
A	+						Excluded
B	-	-	-	-	-	-	HPV negative
C	-	+	-	-	-	-	Incident clearing
D	-	+	-	-	+	-	Incident clearing
E	-	+	-	0	0	0	Incident clearing
F	-	+	-	-	+	+	Incident clearing
G	-	+	-	+	-	+	Excluded
H	-	+	+	-	-	-	Incident persistent
I	-	+	+	-	+	+	Incident persistent
J	-	+	+	+	+	+	Incident persistent
K	-	+	+	-	-	+	Incident persistent
L	-	+	+	0	0	0	Incident persistent

Incidence rates (IR) were calculated for incident clearing (IRI) and persistent (IRP) infections to compare with VL measurements. Person-time for any infection was only counted when at least two subsequent measurements were available after the first HPV TS positive sample. As the exact moment of infection could not be determined, interval censoring was applied for incident clearing infections after the first TS HPV positive measurement. For persistent infections one more sample after the initial TS HPV positive sample was included after which the participant was censored. IRI and IRP were adjusted for factors associated with vaccination status, as reported previously: age, urbanization degree, ever smoked, ever used contraception, ever had sex [12]. Adjusted (a)IRI and aIRP ratios comparing vaccinated and non-vaccinated participants were calculated with 95% confidence intervals (CI) using a Poisson model. Ratios not overlapping unity were considered statistically significant.

Participants supplied samples annually. Participants were excluded from a type-specific (TS) analysis if positive at baseline for that human papillomavirus (HPV) type (A). Following a negative TS HPV baseline measurement, participants were either negative, incident clearing or incident persistent for that HPV type (B-F, H-L). Intermittent infections (G) were excluded due to uncertainty about true incidence or latent persistence. Infections meeting definitions for both clearing and persistent infections were only included based on the first infection identified for that HPV type (F, K).

### Statistical analysis

Statistical analysis was performed using GraphPad Prism 7 (GraphPad Software) and SAS 9.4 (SAS Institute Inc.). Two-tailed p-values <0.05 were considered statistically significant. Differences in study populations for the present analysis and the study population as published by Donken *et al*. [12] were assessed via chi square and student’s t-test. VL differences were initially assessed using the Mann-Whitney U test based on vaccination status for the complete dataset and then stratified for incident and persistent infections.

## Results

### Study characteristics

From a total of 1832 HAVANA study participants, 115 women were excluded due to a missing sample at baseline or incomplete or unknown vaccination status. 1717 non-vaccinated (n = 769; 44.8%) or three-dose vaccinated (n = 948; 55.2%) girls were included in this analysis. Characteristics of the selected study population are listed in Table 2 and did not differ significantly for any of the tested variables when compared to the study population described by Donken *et al*. [12]. In total, 621 incident clearing infections (327 in vaccinated and 294 in non-vaccinated) and 484 incident persistent infections (232 in vaccinated and 252 in non-vaccinated) were identified. The most prevalent HPV types by absolute count in both vaccine and non-vaccine recipients were HPV51 (n = 123; n = 100 respectively), HPV66 (n = 88; n = 65), and HPV52 (n = 59; n = 57), as shown in Tables 3 and 4.

**Table 2:** General characteristics at baseline for the study population subset.

	*Present analysis*	*Donken et al*. [12]	
**Vaccination coverage**			
n (%)	948 (55)	875 (54)	p = 0.32
**Mean age (range)**			
Non-vaccinated	15 (14–17)	15 (14–17)	p = 0.99
Vaccinated	15 (14–16)	15 (14–16)	p = 0.90
**Low urbanisation**			
Non-vaccinated	227 (31)	227 (31)	p = 0.95
Vaccinated	126 (14)	114 (13)	
**Ever smoked**			
Non-vaccinated	249 (38)	248 (38)	p = 0.89
Vaccinated	282 (32)	267 (32)	
**Ever used contraception**			
Non-vaccinated	314 (42)	314 (42)	p = 0.82
Vaccinated	350 (38)	332 (38)	
**Ever had sex**			
Non-vaccinated	210 (28)	210 (28)	p = 0.97
Vaccinated	201 (22)	187 (22)	

**Table 3:** Infection characteristics of the study cohort, stratified by vaccination status and HPV type for incident clearing infections.

	Incident clearing infections									
	Non-vaccinated				Vaccinated				IRI Ratio	
	Cases	PY	IR per 1000 PY	Adjusted	Cases	PY	IR per 1000 PY	Adjusted	Ratio (95%CI)	Adjusted
**HPV6**	35	3490	10.03 (7.20–13.97)	9.04 (6.20–13.17)	39	4153	9.39 (6.86–12.85)	8.24 (5.82–11.67)	0.94 (0.59–1.48)	0.91 (0.55–1.50)
**HPV11**	10	3625	2.76 (1.48–5.13)	2.33 (1.13–4.82)	12	4266	2.81 (1.60–4.95)	2.79 (1.53–5.08)	1.02 (0.44–2.36)	1.20 (0.47–3.02)
**HPV16**	22	3487	6.31 (4.15–9.58)	5.53 (3.37–9.05)	9	4279	2.10 (1.10–4.04)	2.12 (1.08–4.14)	0.33 (0.15–0.72)	**0.38 (0.17–0.87)**
**HPV18**	12	3567	3.37 (1.91–5.93)	1.52 (0.67–3.48)	13	4270	3.05 (1.77–5.24)	1.41 (0.64–3.08)	0.91 (0.41–1.98)	0.92 (0.39–2.21)
**HPV31**	22	3544	6.21 (4.09–9.43)	5.36 (3.24–8.89)	7	4285	1.63 (0.78–3.43)	1.70 (0.80–3.59)	0.26 (0.11–0.62)	**0.32 (0.13–0.76)**
**HPV33**	15	3604	4.16 (2.51–6.91)	3.62 (1.96–6.67)	5	4284	1.17 (0.49–2.80)	1.12 (0.45–2.77)	0.28 (0.10–0.77)	**0.31 (0.11–0.88)**
**HPV35**	5	3642	1.37 (0.57–3.30)	0.60 (0.11–3.20)	0	4317	-	-	-	-
**HPV39**	24	3544	6.78 (4.54–10.10)	5.12 (3.12–8.39)	21	4190	5.01 (3.27–7.69)	3.82 (2.31–6.31)	0.74 (0.41–1.33)	0.75 (0.39–1.41)
**HPV45**	13	3610	3.60 (2.09–6.20)	2.75 (1.33–5.67)	1	4320	0.23 (0.03–1.64)	0.20 (0.03–1.47)	0.06 (0.01–0.49)	**0.07 (0.01–0.56)**
**HPV51**	46	3343	13.76 (10.31–18.37)	10.26 (7.25–14.59)	62	3917	15.83 (12.34–20.30)	14.56 (11.07–19.15)	1.15 (0.79–1.68)	1.42 (0.93–2.16)
**HPV52**	29	3468	8.37 (5.81–12.03)	7.19 (4.71–10.99)	22	4151	5.30 (3.49–8.05)	4.85 (3.09–7.61)	0.63 (0.36–1.10)	0.67 (0.38–1.21)
**HPV56**	30	3502	8.57 (5.99–12.25)	6.85 (4.47–10.52)	36	4160	8.66 (6.24–12.00)	8.46 (5.98–11.96)	1.01 (0.62–1.64)	1.23 (0.72–2.12)
**HPV58**	9	3623	2.48 (1.29–4.77)	1.94 (0.88–4.30)	7	4276	1.64 (0.78–3.43)	1.36 (0.60–3.12)	0.66 (0.25–1.77)	0.70 (0.25–1.95)
**HPV59**	15	3627	4.13 (2.49–6.86)	3.56 (1.98–6.38)	17	4234	4.02 (2.50–6.46)	3.36 (1.96–5.78)	0.97 (0.48–1.94)	0.95 (.45–1.97)
**HPV66**	40	3466	11.54 (8.47–15.73)	10.97 (7.78–15.46)	43	4028	10.68 (7.72–14.39)	9.22 (6.60–12.87)	0.93 (0.60–1.42)	0.84 (0.53–1.34)

Observed person years (PY) are shown, with incidence rates (IR) for incident (IRI) and persistent (IRP) infections. The ratio represents vaccinated IR per 1000PY divided by non-vaccinated IR per 1000PY. Rates were adjusted for age, urbanization degree, ever smoked, ever used contraception and ever had sex according to [12]. Significant results are displayed in bold.

-: No value could be calculated due to zero case incidence.

#: Model did not converge due to insufficient data points.

**Table 4:** Infection characteristics of the study cohort, stratified by vaccination status and HPV type for incident persistent infections.

	Incident persistent infections									
	Non-vaccinated				Vaccinated				IRP Ratio	
	Cases	PY	IR per 1000 PY	Adjusted	Cases	PY	IR per 1000 PY	Adjusted	Ratio (95%CI)	Adjusted
**HPV6**	14	3569	3.92 (2.32–6.62)	3.57 (1.97–6.47)	13	4232	3.07 (1.78–5.29)	2.15 (1.11–4.17)	0.78 (0.37–1.67)	0.60 (0.26–1.40)
**HPV11**	2	3642	0.55 (0.14–2.20)	#	5	4296	1.16 (0.48–2.80)	0.29 (0.04–2.00)	2.12 (0.41–10.92)	#
**HPV16**	33	3588	9.21 (6.54–12.95)	7.70 (5.02–11.82)	3	4291	0.69 (0.23–2.17)	0.62 (0.20–1.97)	0.08 (0.02–0.25)	**0.08 (0.02–0.27)**
**HPV18**	15	3612	4.15 (2.50–6.89)	3.37 (1.76–6.46)	0	4278	-	-	-	**-**
**HPV31**	21	3620	5.80 (3.78–8.90)	5.38 (3.25–8.88)	5	4306	1.16 (0.48–2.79)	0.94 (0.35–2.55)	0.20 (0.08–0.53)	**0.18 (0.06–0.52)**
**HPV33**	5	3642	1.37 (0.57–3.30)	0.86 (0.27–2.74)	5	4308	1.16 (0.48–2.79)	0.96 (0.35–2.62)	0.85 (0.24–2.92)	1.12 (0.26–4.40)
**HPV35**	4	3664	1.09 (0.41–2.91)	0.55 (0.13–2.29)	3	4324	0.69 (0.22–2.15)	0.57 (0.16–2.09)	0.64 (0.14–2.84)	1.05 (0.20–5.57)
**HPV39**	17	3611	4.71 (2.93–7.57)	3.77 (2.12–6.71)	17	4265	3.99 (2.48–6.41)	3.55 (2.09–6.01)	0.85 (0.43–1.66)	0.94 (0.43–2.04)
**HPV45**	3	3643	0.82 (0.27–2.55)	0.00 (0.00–0.02)	1	4324	0.23 (0.03–1.64)	0.00 (0.00–0.01)	0.28 (0.03–2.70)	0.24 (0.03–2.35)
**HPV51**	54	3508	15.39 (11.79–20.10)	14.78 (11.03–19.80)	61	4111	14.84 (11.55–19.07)	14.12 (10.79–18.48)	0.96 (0.67–1.39)	0.96 (0.65–1.41)
**HPV52**	29	3558	8.15 (5.66–11.73)	7.12 (4.68–10.81)	37	4251	8.70 (6.31–12.01)	7.87 (5.52–11.22)	1.07 (0.66–1.74)	1.11 (0.65–1.90)
**HPV56**	24	3595	6.68 (4.48–9.96)	5.02 (3.06–8.23)	19	4255	4.47 (2.85–7.00)	3.65 (2.20–6.07)	0.67 (0.37–1.22)	0.73 (0.38–1.40)
**HPV58**	5	3650	1.37 (0.57–3.29)	0.52 (0.13–1.99)	7	4306	1.62 (0.78–3.41)	0.66 (0.20–2.20)	1.19 (0.38–3.74)	1.28 (0.37–4.44)
**HPV59**	2	3658	0.55 (0.14–2.19)	0.25 (0.03–1.86)	11	4276	2.57 (1.43–4.65)	2.12 (0.10–4.37)	4.71 (1.04–21.23)	**8.57 (1.06–69.04)**
**HPV66**	25	3582	6.98 (4.72–10.33)	6.82 (4.46–10.41)	45	4173	10.78 (8.05–14.44)	10.00 (7.26–13.77)	1.55 (0.95–2.52)	1.47 (0.87–2.48)

Observed person years (PY) are shown, with incidence rates (IR) for incident (IRI) and persistent (IRP) infections. The ratio represents vaccinated IR per 1000PY divided by non-vaccinated IR per 1000PY. Rates were adjusted for age, urbanization degree, ever smoked, ever used contraception and ever had sex according to [12]. Significant results are displayed in bold.

-: No value could be calculated due to zero case incidence.

#: Model did not converge due to insufficient data points.

The present analysis was compared to a previous epidemiological description of this cohort study [12] and differences were assessed.

### HPV16/18 vaccination leads to reduced incidence of HPV16/18, with some cross-protective effects

Incidence rates and ratios were calculated per thousand observed person years for vaccinated and non-vaccinated individuals and adjusted according to Donken *et al*. [12], as shown in Tables 3 and 4. In line with results reported by Donken *et al*. [12], we find significantly less incident clearing and persistent HPV16 infections in the vaccinated group (aIRI ratio: 0.38; 95%CI: 0.17–0.87; aIRP ratio: 0.08; 95%CI: 0.02–0.27). For HPV18 no significant effect was observed against clearing infections, but persistent infections did not occur at all in vaccinated individuals (no ratio calculated, vaccinated IRP of zero).

For non-vaccine types, a reduced incidence of incident clearing infections was found in the vaccinated group for HPV31 (aIRI ratio: 0.32, 95%CI: 0.13–0.76), HPV33 (aIRI ratio: 0.31, 95%CI: 0.11–0.88), HPV35 (no ratio calculated, vaccinated aIRI of zero) and HPV45 (aIRI ratio: 0.07, 95%CI: 0.01–0.56). Additionally, less HPV31 persistent infections were found among vaccinated study participants (aIRP ratio: 0.18, 95%CI: 0.06–0.52). Interestingly, more HPV59 persistent infections were found in the vaccinated group (aIRP ratio: 8.57, 95%CI: 1.06–69.04). For other HPV types, no (significant) differences were observed.

### Type-specific quantification of HPV viral load

**Fig 1:**
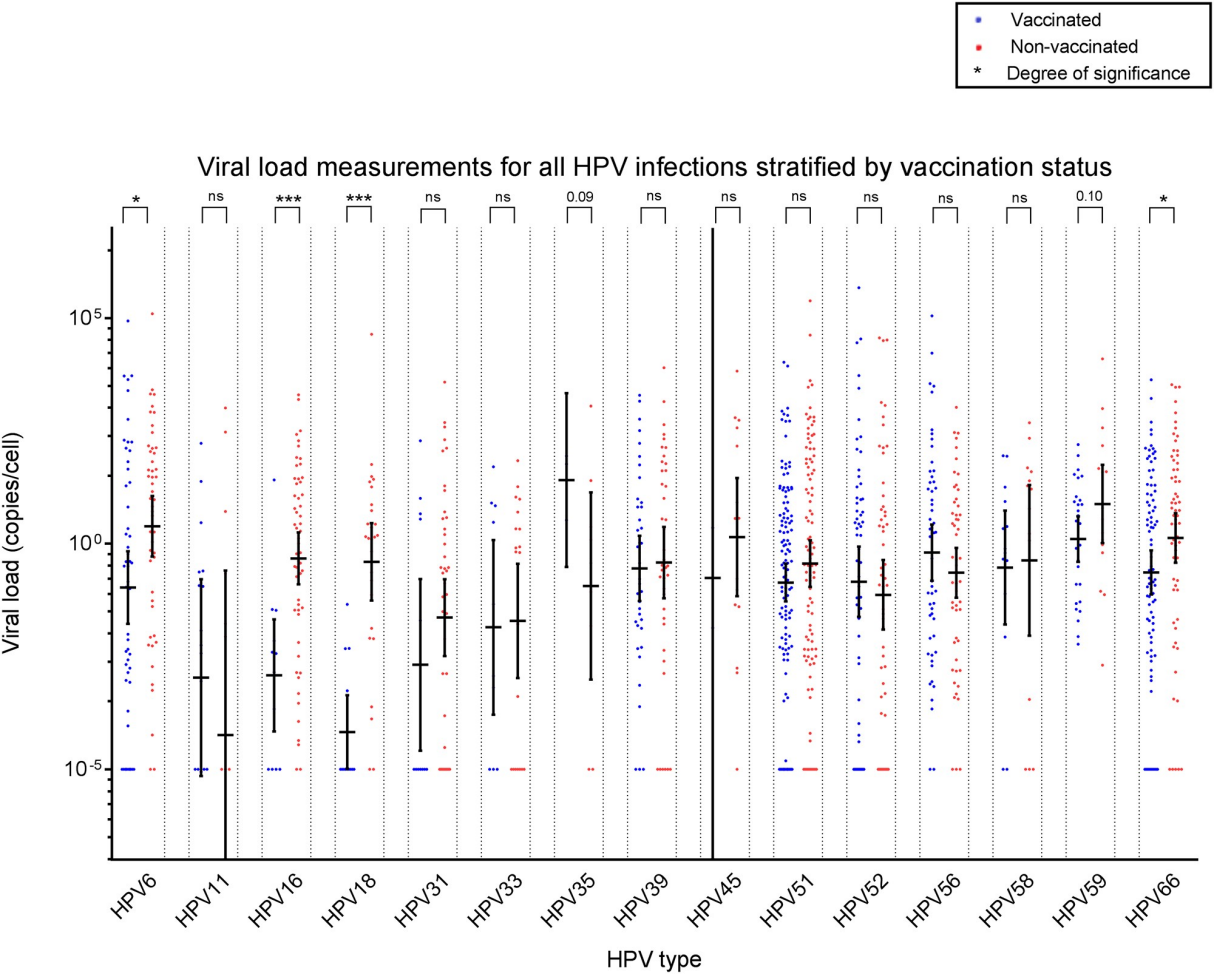
Viral load measurements of all infections included in this study, expressed in copies per cell on a log10 scale. Individual measurements are displayed as blue and red dots for vaccinated and non-vaccinated study participants respectively. For each HPV type median VL values were compared between vaccinated and non-vaccinated individuals. Statistical significance is shown above each HPV type, with * meaning p<0.05; ** meaning p<0.01; *** meaning p<0.001.

**Fig 2:**
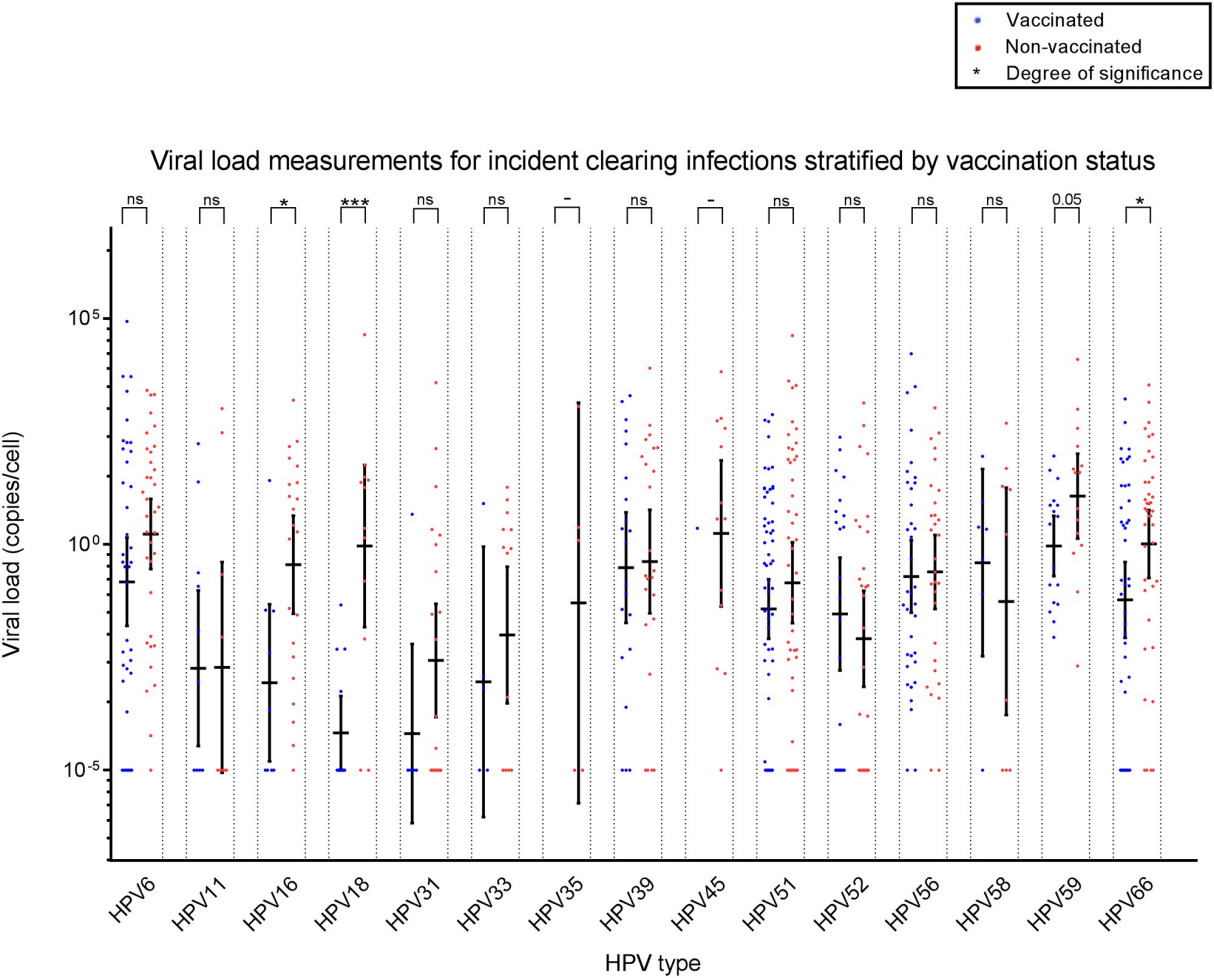
Viral load measurements in incident infections, expressed in copies per cell on a log10 scale. Individual measurements are displayed as blue and red dots for vaccinated and non-vaccinated study participants respectively. For each HPV type median VL values were compared between vaccinated and non-vaccinated individuals. Statistical significance is shown above each HPV type, with * meaning p<0.05; ** meaning p<0.01; *** meaning p<0.001.

**Fig 3:**
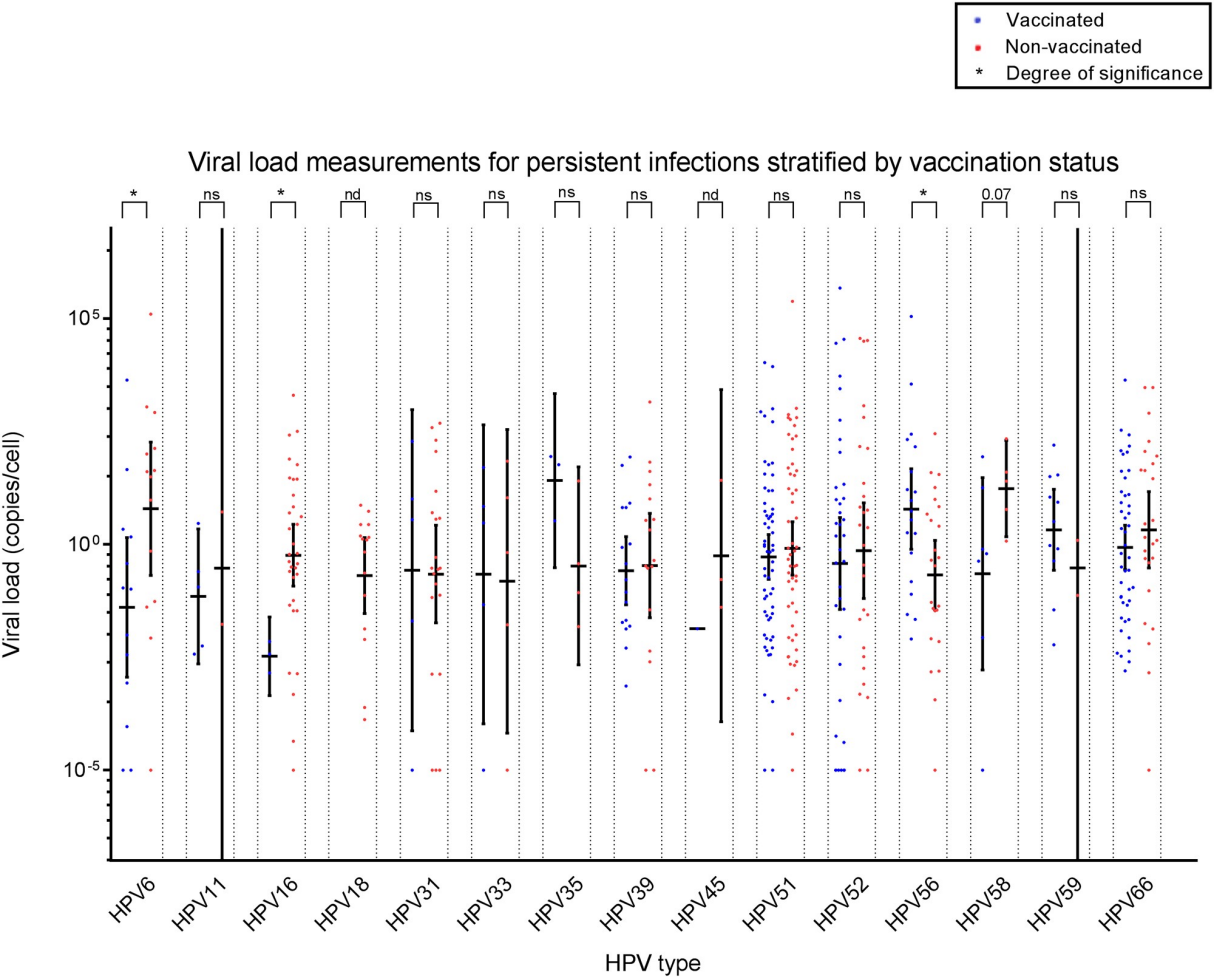
Viral load measurements in persistent infections, expressed in copies per cell on a log10 scale. Individual measurements are displayed as blue and red dots for vaccinated and non-vaccinated study participants respectively. For each HPV type median VL values were compared between vaccinated and non-vaccinated individuals. Statistical significance is shown above each HPV type, with * meaning p<0.05; ** meaning p<0.01; *** meaning p<0.001.

HPV6, 11, 31, 33, 35, 39, 45, 51, 52, 56, 58, 59 and 66 TS VL assays were specific in the presence of at least 10^7^ copies of different HPV types. Sensitivity was <5 GEq/reaction for each individual assay. Reproducibility of VL measurements was assessed by measuring duplicates of a subset of HPV16 (n = 62) and HPV18 (n = 22) samples. Duplicates were found to be on average <0.1 ct values apart (median 0.03; minimum -2.39; maximum 1.66). VL measurements were performed based on genotyping results. Figs 1–3 show scatter plots of VL measurements for study participants in all infections, the clearing infections subset, and persistent infections subset respectively.

### Reduced viral load in breakthrough HPV16/18 infections following HPV16/18 vaccination

VL measurements correlated well with observed reductions in incident/persistent HPV16/18 infections. Median HPV16 VL was significantly lower in vaccine recipients for the total study analysis (p<0.01, Fig 1), as well as stratified analyses for incident clearing (p = 0.02, Fig 2) and persistent infections (p = 0.05, Fig 3). For HPV18, vaccine recipients showed significantly lower median VL in the total analysis (p<0.01, Fig 1) and in the incident clearing analysis (p<0.01, Fig 2). Since no persistent HPV18 infections were identified among vaccinees, no VL comparison could be performed.

### Cross-protective effects on viral load following vaccination are unclear

Despite finding some cross-protection of the bivalent vaccine against HPV31, 33, 35 and 45 (Tables 3 and 4), no significant differences in median VL were observed between groups in any of the analyses for these types. Except for a marginally increased median HPV35 VL in the vaccinated group for the total analysis (p = 0.09), although this was based on a limited number of infections (n = 3 vaccinated, n = 9 non-vaccinated). For HPV6, significantly lower median VL values were found in the vaccinated group for the total (p = 0.01) and the persistent infections analyses (p = 0.05). For HPV59 and 66 (borderline) lower median VL values were found in the vaccinated group for the total (p = 0.10; p = 0.03) and incident clearing (p = 0.05; p = 0.04) infection analyses. For HPV58 a marginally lower median VL was observed in vaccinated individuals (p = 0.07). For HPV56 an increased median VL was observed in the vaccinated group (p = 0.03). The VL results for HPV6, 56, 58, 59 and 66 did not lead to a measurable decrease in the rate of incident or persistent infections in the vaccinated group. Interestingly, the median VL of any HPV type in the vaccinated group shows a trend in being slightly lower than in the non-vaccinated group (Figs 1–3). To indicate compatibility between incidence and VL analyses, Table 5 shows summarized results per HPV type, which shows good concordance between analyses for vaccine types, but poor concordance for non-vaccine types.

**Table 5:** Summary and compatibility of HPV infection incidence with viral load.

	Vaccinated compared to non-vaccinated groups; effects measured on:				
	Infection incidence rate		Viral load		
HPV type	Incident clearing	Incident persistent	Total infections	Clearing infections	Persistent infections
6	-	-	Reduced	-	Reduced
11	-	-	-	-	-
16	Reduced	Reduced	Reduced	Reduced	Reduced
18	-	Reduced ^1^	Reduced	Reduced	- ^1^
31	Reduced	Reduced	-	-	-
33	Reduced	-	-	-	-
35	Reduced ^1^	-	Increased ^2^	- ^1^	-
39	-	-	Reduced ^2^	-	-
45	Reduced	-	-	- ^1^	-
51	-	-	-	-	-
52	-	-	-	-	-
56	-	-	-	-	Increased
58	-	-	-	-	Reduced ^2^
59	-	Increased	Reduced ^2^	Reduced	-
66	-	-	Reduced	Reduced	-

Reduced/increased incidence or viral load implies a reduction in the vaccinated group compared to the non-vaccinated group.

^1^: Calculation for p-value could not be performed due to complete absence of infections in the vaccinated group.

^2^: Borderline significant results (0.05 < p <0.1).

## Discussion

In this study, newly developed and existing assays [26] were used to measure VL of HPV6, 11, 16, 18, 31, 33, 35, 39, 45, 51, 52, 56, 58, 59 and 66 infections on a prospective longitudinal observational cohort study of young women who were eligible for HPV vaccination according to a three-dose schedule. To our knowledge, we are the first to study effects of HPV vaccination on the VL of HPV infections. We confirm that vaccination with the bivalent vaccine leads to significant reductions in the incidence of clearing and/or persistence of HPV16/18 infections, in line with previous results for a different sample subset of this study by Donken *et al*. [12]. In addition, we show that vaccination is associated with significant reductions in median VL of breakthrough incident clearing and persistent HPV16/18 infections. Vaccination with the bivalent vaccine also induced a reduction in both incident clearing and persistent HPV31 infections as well as reductions in incident clearing HPV33 and 45 infections. However, these findings were not associated with any reductions in VL of these HPV types following vaccination. Our findings suggest that vaccination is significantly associated with reduction in VL in breakthrough vaccine type infections, possibly by limiting the capacity of the virus to cause a persistent infection. However, despite confirming cross-protection against HPV31, 33 and 45, the effect on VL is not significantly altered, suggesting that cross-protection against non-vaccine HPV types is either complete (preventing infections in a similar fashion to vaccine types) or absent (not measurable by VL in this study).

Cross-protection has been previously described for HPV31, 33, 35, 45, 52 and 58 [7–10]. We could confirm some of these effects in this study (HPV31, 33, 35 and 45), but no significant VL reductions in the vaccinated group were found for either type, despite a reduced effect, although HPV33 and 45 infections are uncommon in this study (n = 10 for HPV33, n = 4 for HPV45). For HPV6, 59 and 66 (borderline) significant median VL reductions were found among vaccine recipients, although this did not translate into any measurable reduction of incident clearing or persistent infections for these HPV types in this study. Cross-protection is less likely against HPV6 and 66, as they belong to a different HPV species than HPV16 or 18 [35]. For HPV59, despite it being closely related to HPV18, the SPF10-DEIA-LiPA_25_ genotyping assay has a relatively poor detection sensitivity [36], which might explain the lack of concordance between HPV59 VL and incidence results.

Vaccination (with any of the currently available virus-like particle (VLP) based vaccines) primarily leads to sterilizing immunity against HPV16/18 via type-restricted neutralizing antibodies, preventing the majority of incident and persistent infections [37]. We report significantly lower median VL values in vaccinated individuals with HPV16/18 breakthrough infections. Our results could suggest that the immune response protecting against HPV infection acquisition could be different from the immune response responsible for viral control. This effect might be facilitated by a T cell mediated immune response limiting viral replication, which is the proposed mechanism leading to cross-protection against non-vaccine type associated CIN2+ [37, 38], and which could help explain results described by Harper *et al*. [16]. This could result in infections with lower VL levels, which have been associated with clearance of infection [39, 40], while high HPV16/18 VL levels have been associated with persisting infections [25, 26].

While we are unable to clearly associate vaccine effects on incidence of infections with VL, we do observe a general trend where median VL for any HPV type was lower for vaccine recipients than non-vaccinated individuals. If these findings are indeed caused by the vaccine, they might help alleviate or prevent long-term disease effects by dampening infection development through reduced viral load.

Several limitations need to be addressed for this study. First, we included participants based on baseline TS HPV DNA negative samples, since serology was only available for a subset of our cohort. While baseline DNA positivity is informative for the present sample, it does not inform about any past exposure to HPV and any possible effects on VL. Fortunately, due to low sexual activity at inclusion for this study (Table 2), we believe prior infections should be of limited impact for the present analysis. Second, our categorical definitions of incident clearing and persistent infections (Table 1), though pragmatic and in line with previous literature use, might not accurately represent a natural situation and do not take possible deposition of HPV into account [41]. Incident persistent infections should suffer less from HPV depositions, as repeated detection of deposition events one year apart seems unlikely. Based on a previous study describing TS clearance windows for individual HPV types, one year for persistent infections is adequate in general [42]. Ideally, longer intervals should be observed before infections can be truly considered persistent. However, within this study of young, vaccinated women, infections are relatively scarce and longer interval persistent infections would lead to sharp reductions in incidence, prohibiting statistical interpretation of results. Our definitions also lead to exclusion of intermittent infections. For intermittence, it is unclear whether the observed infection is actually persistent with potential latency in between, or actual a repeated incidence of the same genotype. Since, previous research has shown that relative risk for repeat infections might be different from initial acquisition, these infections were excluded [43]. Third, VL is a highly heterogeneous parameter [26, 44]. Combined with a relatively low number of total infections in this study, this could explain why VL point estimates (Figs 1–3) are often lower in the vaccinated group than in the non-vaccinated group; yet no statistical significance is reached in comparisons and compatibility between incidence rates and VL results is low (Table 5), except for vaccine types HPV16/18. Finally, during VL measurements, a number of samples remained VL negative, despite positive genotyping results. For these samples, an artificial VL was assigned [33]. This method was chosen over assigning a VL at the detection limit of the HPV type tested, since this would lead to exaggerated results when correcting for cellular content.

## Conclusions

Combined, our results suggest that vaccination against HPV16/18 affects VL in breakthrough infections when compared to non-vaccinated women. Although some effects are observed against non-vaccine types, this study is insufficiently powered to suggest a clear correlation between vaccination and effects on VL of non-vaccine HPV types. Further population-based studies are required to identify which effects are truly causal to and maintained by the vaccine.

## Supporting information

S1 Table Sequences of primers and probes used for the quantification of different HPV types and limits of detection (LOD) of respective assays. (DOCX)
