# Supplementary material for: Effect of the bivalent HPV vaccine on viral load of vaccine and non-vaccine HPV types in incident clearing and persistent infections in young Dutch females
Source: PLoS One. 2019 Mar 4;14(3):e0212927. doi: 10.1371/journal.pone.0212927 (PMC6398842; doi:10.1371/journal.pone.0212927)
Supplement: S1 Table — (DOCX) [file pone.0212927.s001.docx]

S1 Table: Sequences of primers and probes used for the quantification of different HPV types and limits of detection (LOD) of respective assays.

| **Genotype** | **Forward (5'-3')** | **Reverse (5'-3')** | **Fluorophore (5')** | **Probe (5'-3')** | **Quencher (3')** | **LOD**  **Copies/reaction** |  |  |  |  |  |
| --- | --- | --- | --- | --- | --- | --- | --- | --- | --- | --- | --- |
| HPV6 | CAATGGTATTTGTTGGGGTAATCAAC | TGTGGAAGATGTAGTTACGGATGCACA | FAM | ACCACACGCAGTACCAACATGACCA | EDQ | 1.8 |  |  |  |  |  |
| HPV11 | AGGTAGGCAGGGGTCAACCT | CACAGCGTTTAGTATGGGCGTGCACA | FAM | CCAGGATTACCACCATACCCACCA | EDQ | 2.6 |  |  |  |  |  |
| HPV16 | TTGTTGGGGTAACCAACTATTTGTTACTGTT | CCTCCCCATGTCTGAGGTACTCCTTAAAG | FAM | TGTCATTATGTGCTGCCATATCTACTTC | TAMRA | 4.6 |  |  |  |  |  |
| HPV18 | GCATAATCAATTATTTGTTACTGTGGTAGATACCACT | GCTATACTGCTTAAATTTGGTAGCATCATATTGC | Yakima Yellow | AACAATATGTGCTTCTACACAGTCTCCTGT | BHQ-2 | 4.3 |  |  |  |  |  |
| HPV31 | GGTAGATACCACACGTAGTACCAATATGTCTGTTTG | AAATTGTAAATCAAATTCCTCACCATGTCTTAAATACTCT | FAM | TGCTGCAATTGCAAACAGTGATACTACATT | EDQ | 1.2 |  |  |  |  |  |
| HPV33 | GGAACTACTGCCTCTATTCAAAGCAGTGC | GTACTGTCACTAGTTACTTGTGTGCATAAAGTCATA | FAM | CTGTGGTAGATACCACTCGCAGTACTAAT | EDQ | 1.0 |  |  |  |  |  |
| HPV35 | AAAAGGCACACCTTGTAATGCTAACCAG | AATCCATTGCACCAAATCCTGTGTC | FAM | TGTACTACAAGACGGGGACATGGT | EDQ | 1.2 |  |  |  |  |  |
| HPV39 | CGTGGTGGTATGGTGGGTGACGCCAT | ATGGAACCGCTGGGAGAGGGGCAGTATACA | FAM | CGTGCAAACCCCGGTAGTTC | EDQ | 1.4 |  |  |  |  |  |
| HPV45 | TAGTGGACACTACCCGCAGTACTAA | CCACATGTCTACTATACTGCTTAAACTTAGTAG | FAM | ACACAAAATCCTGTGCCAAGTACATATGAC | EDQ | 1.8 |  |  |  |  |  |
| HPV51 | AACAATCAGCTTTTTATTACCTGTGTTGATACTACCA | CTTAAAGTTACTTGGAGTAAATGTTGGGGAAACCG | FAM | CAAATTTAACTATTAGCACTGCCACTGCT | EDQ | 1.5 |  |  |  |  |  |
| HPV52 | TTGGGGCAATCAGTTGTTTGTCACAGTT | CGCCATGACGAAGGTATTCCTTAAAATTTTCATTTT | FAM | TTATGTGCTGAGGTTAAAAAGGAAAGCACA | EDQ | 1.8 |  |  |  |  |  |
| HPV56 | CCCGCTATGGGTGAACATTGGA | AATGCAAGAGGCGGGCAGTC | FAM | AGGTGCTGTGTGTAAGTCCACACAA | EDQ | 1.2 |  |  |  |  |  |
| HPV58 | CAGTTATTTGTTACCGTGGTTGATACCACTCG | TGTAAGTCATATTCTTCAACATGACGTACATATTCC | FAM | TGCACTGAAGTAACTAAGGAAGGTACA | EDQ | 1.6 |  |  |  |  |  |
| HPV59 | CCACTATTTTGGAGGATTGGAATTTTGG | GCGGTGCGGTGTCCTTTTGA | FAM | CCGTTTTGTTCAATCTGCTGCTGT | EDQ | 1.2 |  |  |  |  |  |
| HPV66 | GCACATTAACTAAATATGATGCCCGTGAA | AAGTTGAAACACAAACTGTAGTTCATATTCCTCCA | FAM | ATCAATCAATACCTTCGCCATG | EDQ | 1.4 |  |  |  |  |  |
